# Registered nurses’ experiences of providing respiratory care in relation to hospital- acquired pneumonia at in-patient stroke units: a qualitative descriptive study

**DOI:** 10.1186/s12912-020-00518-7

**Published:** 2020-12-20

**Authors:** Gunilla Borglin, Miia Eriksson, Madeleine Rosén, Malin Axelsson

**Affiliations:** 1 Department of Care Science, Faculty of Health and Society, Malmö University, SE-205 06 Malmö, Sweden; 2 Department of Nursing Education, Lovisenberg Diaconal University College, 0456 Oslo, Norway; 3 Department of Neurology, Skåne University Hospital, SE-222 42 Malmö, Sweden

**Keywords:** Content analysis, Fundamentals of care, Nursing, Qualitative research, Stroke care

## Abstract

**Objective:**

This study aimed to describe registered nurses’ (RNs) experiences of providing respiratory care in relation to hospital acquired pneumonia (HAP), specifically among patients with acute stroke being cared for at in-patient stroke units.

**Background:**

One of the most common and serious respiratory complications associated with acute stroke is HAP. Respiratory care is among the fundamentals of patient care, and thus competency in this field is expected as part of nursing training. However, there is a paucity of literature detailing RNs’ experiences with respiratory care in relation to HAP, specifically among patients with acute stroke, in the context of stroke units. As such, there is a need to expand the knowledge base relating to respiratory care focusing on HAP, to assist with evidence-based nursing.

**Design:**

A qualitative descriptive study.

**Method:**

Eleven RNs working in four different acute stroke units in Southern Sweden participated in the current study. The data were collected through semi-structured interviews, and the transcribed interviews were analysed using inductive content analysis.

**Results:**

Three overarching categories were identified: (1), awareness of risk assessments and risk factors for HAP (2) targeting HAP through multiple nursing care actions, and (3) challenges in providing respiratory care to patients in risk of HAP. These reflected the similarities and differences in the experiences that RNs had with providing respiratory care in relation to HAP among in-patients with acute stroke.

**Conclusions:**

The findings from this study suggest that the RNs experience organisational challenges in providing respiratory care for HAP among patients with acute stroke. Respiratory care plays a vital role in the identification and prevention of HAP, but our findings imply that RNs’ knowledge needs to be improved, the fundamentals of nursing care need to be prioritised, and evidence-based guidelines must be implemented. RNs would also benefit from further education and support, in order to lead point-of-care nursing in multidisciplinary stroke teams.

## Background

One of the most common and serious respiratory complications associated with acute stroke is hospital acquired pneumonia (HAP). Several terms are used in the literature to refer to pneumonia following an acute stroke. In this study, the term hospital acquired pneumonia (HAP) will be used, per recommendation from the European Stroke Organisation, (ESO) [1]. The ESO defines HAP as an acute lower respiratory tract infection, acquired after a minimum of 48 h of hospitalisation, and not having been incubating at the time of admission [1]. The incidence of HAP in stroke units is estimated to be between 39 and 44% [2]. Among stroke patients, the mortality rate from pneumonia is higher than that for other care-related infections, such as urinary tract infections. Recovery from an acute stroke has also been found to be negatively impacted if, post stroke, the patient acquires HAP as it increases the likelihood of dependency [3] and the time in care by up to 7.5 days [4]. This is significant, as around 15 million people worldwide suffer from stroke annually [5], underscoring the importance of improving knowledge relating to respiratory nursing care, especially to prevent and target complications such as pneumonia among this substantial group of patients.

Nowadays, the majority of RNs working with stroke patients are doing so at an organised in-patient unit (stroke unit) which is acknowledged as an evidence-based model of patient management [6]. At these units’ the RNs are part of a co-ordinated teamwork, which requires that a certain percentage of the team has completed specialist stroke training (RNs included), and care is expected to be based on evidence, in addition to safety standards [6]. Within the team, RNs are the largest professional group [7], and are described as those with the most prolonged contact with patients, i.e., 24 h a day [8]. As a result, RNs are expected to possess the knowledge and skills needed to provide the patients with excellent care at these units. The role of an RN is to assess, diagnose, intervene, and evaluate the patient’s fundamental personal needs [9]. Richards and Borglin [10] point out that one of the crucial elements of nursing, should be that they are focused on the patients’ essential personal needs, including respiration, mobility, cleanliness, and nutrition. These needs, or fundamentals of care, have been rearticulated by Kitson and colleagues [11], to link the psychosocial, physical, and relational dimensions of the nursing care encounter. Respiratory care includes the assessment of breathing (respiration), to identify problems and complications early on, and is a vital part of the fundamentals of care [11], and thus competency in this field is a standard requirement of RNs. Among acute stroke patients, it is particularly important that their RNs are able to identify and address respiratory complications. Specifically, by identifying patients at risk of respiratory problems, whilst simultaneously delivering relevant nursing care to those who have already developed such problems, RNs help to prevent further complications from developing, including pneumonia, which is one of the leading causes of death for stroke patients [12] However, published explorations of what actually constitutes respiratory nursing care in practise, are sparse, especially in the context of stroke units. Additionally, how nursing care in such dedicated units, and as a part of a co-ordinated teamwork influences the delivery of nursing care is neither well described. Nonetheless, it is evident that being cared for at an organised in-patient stroke unit are beneficial for this group of patient’s rehabilitation and recovery. In a recent *Cochrane* review*,* it was concluded that one-year post stroke, those patients receiving care at stroke units were more likely than those receiving care outside such units to be alive, be independent, and live at home, i.e., to recover [13]. Working in interprofessional teams to certify patient safety and quality of care, is one of the six core competencies required of RNs to meet health care standards [14], and is a realistic approach to improving nursing care targeting HAP. Indeed, skilled nursing care has been proposed as an essential component of stroke units [15].

Despite the aforementioned, there is a lack of literature relating to RNs’ experiences of respiratory care focussing on HAP in patients with acute stroke, specifically in the context of stroke units. However, one systematic review taking on a meta-ethnographic approach [16] were identified. Clarke investigated nursing practices in the care and rehabilitation of in-patient stroke survivors, and found that RNs mainly focused on monitoring, and their involvement in post stroke rehabilitation was minimal [16]. Research examining respiratory nursing care relating to pneumonia appears to be mainly quantitative in design, targeting ventilator associated pneumonia (VAP) and in the setting of intensive care units [17]. The scarcity of literature focussing on RNs’ professional role in preventing HAP is noteworthy, especially considering its high prevalence among patients following acute stroke [3]. Such knowledge could enhance safe practice, and facilitate the delivery of improved evidence-based respiratory care for stroke patients. Consequently, this study aimed to describe RNs’ experiences of providing respiratory care in relation to HAP, specifically among patients with acute stroke being cared for at in-patient stroke units.

## Methods

A qualitative descriptive design, as per Sandelowski [18], was adopted to understand RNs’ experiences in relation to the study aim. The data were collected through semi-structured interviews as proposed by Polit and Beck [19], and were analysed using inductive content analysis [20, 21]. This study is reported in accordance with the consolidated criteria for reporting qualitative studies – COREQ [22] (Supplementary file 1).

### Setting and participants

This study was conducted across four acute stroke units in the south of Sweden. These stroke units were selected according to the Stroke Unit Trialists’ [6] definition, i.e., stroke units specialising in caring for stroke in-patients where nurses, together with other health care professionals, are working as a coordinated team. A purposive sampling technique was used [23], with eligible participants being RNs who had worked for at least 1 year in one of the four stroke units included in the study.

Information detailing the study was sent via email to the operational managers of each of the stroke units. A week after the initial contact, the operational managers were contacted via telephone. Eleven RNs agreed to participate in the study. Both verbally, and in writing, the participants were informed about the study and the researchers’ objectives, told that their information would be kept confidential, and assured that they could withdraw from the study at any time. Additionally, both oral and written informed consent was given by participants before the interviews started. Ten of the 11 participants were female, and they had all worked as RNs for 2–25 years. Their work experience ranged from 2 to 21 years. Two RNs had undergone specialised education, one being an RN in medicine, the other a district RN, while the rest each had a Bachelor of Nursing Science degree. Six had attended specific courses in stroke care.

### Data collection

The data for this study were collected through semi-structured interviews [19] over 5 weeks, in December 2018, and January 2019. The participants decided on the time and place of the interviews. They all chose their workplace as the venue for the interviews. In total, 13 interviews were conducted. Before data collection, two initial interviews were conducted to assess the quality of the semi-structured interview procedure, resulting in minor rephrasing to facilitate participants understanding. These interviews were not included in the study. Two researchers (MR and ME) conducted the interviews in Swedish, alternating as either an assistant taking notes on any non-verbal communication, or as an interviewer using an interview guide (Table 1). Probing questions (e.g., “Can you please explain further?”, “Can you please give an example?”, and “Can you elaborate?”) were used to encourage participants to elaborate on their responses [19]. After nine interviews, no new information was provided; nevertheless, two more interviews were conducted [24]. The interviews lasted approximately 30–40 min each, and were recorded digitally. They were then transcribed verbatim by the second and third authors (MR and ME).

**Table 1:** Semi-structured interview guide

As a RN with the primary responsibility for patients suffering from acute stroke admitted to the unit*:*	
• What are your experiences of nursing care for this group of patients?	
• In your experience, which specific nursing care needs, in relation to other patient groups, do this group of patients have?	
• In your experience which are the most common complications among this group of patients?	
A) If hospital acquired pneumonia is described probe further in relation to nursing care for this group of patients. B) If the RN not give hospital acquired pneumonia as one of the most common complication in this group ask specifically:	
• What are your spontaneous thoughts about hospital acquired pneumonia in relation to this group of patients?	
• What are your experiences of working with preventative nursing care in regards to hospital acquired pneumonia?	
• What type of nursing interventions do you think could be implemented to prevent hospital acquired pneumonia?	

### Data analysis

The transcripts were analysed according to Graneheim and Lundman’s [20] description of content analysis. According to Krippendorff [23] content analysis constitutes a departure from an inductive methodological approach. The iterative process of analysis took place in several steps. In the first step, the transcribed texts were read repeatedly to obtain an overall understanding [20]. In the second step, key issues (i.e., units of meaning) reflecting the aim of the study were independently highlighted with different coloured pens. Meaning units were then condensed to reflect their essence, after which they were colour coded. Next, the different coloured codes were explored and compared to identify differences and similarities. Finally, they were sorted into tentative subcategories, ensuring that the original content was preserved. While the second and the third authors (MR and ME) oversaw this analysis, all the authors conducted the process described above. The team then met several times to discuss the texts, in order to identify the most likely meaning, whilst maintaining the integrity of the content. This resulted in the authors agreeing upon 11 subcategories (Table 2).

**Table 2:** Overview subcategories and categories

Subcategories	Categories
• Acknowledged risk factors	Awareness of risk assessments and risk factors for HAP
• Taking advantage of routine risk assessments	
• Respiratory care	Targeting HAP through multiple nursing care actions
• Mobility care	
• Oral care	
• Nutritional care	
• Interprofessional teamwork	
• Not part of standard nursing care	Challenges in providing respiratory care to patients in risk of HAP
• Not a prioritised part of nursing care	
• Contextual challenges	
• Environmental context	
• Competence and knowledge level	

In the final step, three overarching categories unifying the content of the 11 subcategories were formulated. These three categories were interpreted to reflect the variations in the RNs’ experiences of providing respiratory care in relation to HAP among in-patients with acute stroke.

## Results

The RNs experiences of providing respiratory care for HAP among in-patients with acute stroke could be understood in light of three overarching categories, (1) awareness of risk assessments and risk factors for HAP, (2) targeting HAP through multiple nursing care actions, and (3) challenges in providing respiratory care to patients in risk of HAP. In this section, the categories are exemplified by quotations (Q) (Tables 3–5), and sub categories are presented in italic.

### Awareness of risk assessments and risk factors for HAP

In the category ‘awareness of risk assessments and risk factors for HAP’, the participants *acknowledged the need to know the range of different risk factors* for developing HAP in patients following acute stroke. These factors included dysphagia, immobility, nasogastric feeding, and other underlying conditions aside from acute stroke itself. In this category, the possibility and importance of *taking advantage of routine care assessments* to identify risks of adverse health care events were also addressed, as was the need for specialist training in the identification of HAP in stroke patients.

The participants described dysphagia as one of the most common risk factors for stroke patients in developing HAP. Therefore, as a part of standard care, some participants described that it was important for routine risk assessments of a patient’s swallowing function always to be undertaken as per Riksstroke [i.e. the Swedish stroke register aiming to ensure continuous quality improvement in Swedish stroke care] (Table 3, Q1). The national guidelines for stroke care state that a swallowing assessment should precede any oral intake of food or drinks. Different experiences were described in terms of how well this routine risk assessment of swallowing function was implemented. Some participants found that all patients admitted to their unit with a diagnosis of acute stroke received an accurate assessment of swallowing function, while others suggested that there was room for improvement in conducting these assessments (Table 3, Q2). The participants also stated that bedridden patients, or patients using a nasogastric feeding tube (NGFT), had a higher risk of developing HAP than mobile patients (Table 3, Q3), or patients without an NGFT. The latter was stated as a substantial risk factor in developing HAP, particularly in those cases where patients were confused, or were not correctly supported or positioned in bed during feeding (Table 3, Q4).

**Table 3:** The category, Awareness of risk assessments and risk factors for HAP

“We are always very particular so that no patient eats or drinks before we have assessed swallowing function, and then we have a schedule we use.” (Q1, RN/E)	
“…Even if we should do [a swallow assessment], it might fail. We could be more meticulous and gain a higher level of competence.” (Q2, RN/F)	
“Yeah, that one does not ventilate the lungs properly. Thus, one does not get the same ventilation [of the lungs] as when one is up and about. So, it becomes very likely that the lung collapses and, simultaneously, the patient maybe swallows some saliva or something, so it is very easy.” (Q3, RN/E)	
“Some of our patients can pull on the nasogastric feeding tube, and it can then end up in the lungs [instead of the oesophagus], or if they are positioned flatly in bed, or [the feeding] goes too fast, and they are positioned on their left side, so the patient could also end up with some pneumonia if it goes wrong or if they aspirate.” (Q4, RN/D)	
“… it is more if they have any other underlying diagnoses, but if they have asthma or COPD, then pneumonia arrives directly with the post man.” (Q5, RN/I)	
“One could maybe identify patients at risk [for pneumonia] … and decide upon a goal for what those patients should do daily to minimise the risk. Just like in the way we identify those patients at risk for fall and pressure ulcers, so maybe one could identify those at risk to develop pneumonia…” (Q6, RN/E)	
“If we consider our assessment measures, we think a lot about risk of falls and pressure ulcers and so on, and it is the first priority, but then one realises after a while, oh, there are maybe more risks, but the risk of attracting pneumonia is not the first thing on my mind when I receive the patient, no it is not.” (Q7, RN/G)	
’Many years ago, it was mandatory to document in Riksstroke if the patient acquired pneumonia. It is not done anymore… it was an eye opener because we saw that we had a lot or… but an increased number got pneumonia, and we started to ransack ourselves. We became good at swallow assessments, and this was one way to raise awareness of the problem [of pneumonia]. We managed to decrease the numbers of pneumonia when we became aware of what we were doing’. (Q8, RN/F)	
“… I have been where there is stroke-educated staff; it is much easier to discover and discuss and to initiate actions… it can always be done better.” (Q9, RN/H)	

The participants also stated that patients who had other underlying conditions, for example patients with chronic obstructive pulmonary disease (COPD), or asthma, were especially vulnerable, and had an increased risk of developing HAP after an acute stroke (Table 3, Q5). This category also reflected an awareness among the participants that respiratory nursing care per se, as well as the assessment and identification of those patients with an increased risk of developing HAP, required the same assessment and identification as the mandatory quality indicators stipulated by Riksstroke (Table 3, Q6). However, some participants described that this strict focus on mandatory quality indicators in nursing care could in fact work against the identification of those most at risk of developing HAP after an acute stroke (Table 3, Q7). On the other hand, other participants expressed a wish to reinstate the quality assurance used in Riksstroke for pneumonia, as part of standard care. They stated that such an action would automatically result in the return of standard care risk assessments for pneumonia. Participants described how this formerly mandatory requirement had increased their awareness and competence as RNs, especially in regard to HAP (Table 3, Q7). Additionally, participants experienced higher preparedness for identifying HAP in stroke units, where staff had attended a course specifically focusing on patients who had suffered a stroke. (Table 3, Q8).

### Targeting HAP through multiple nursing care actions

In the category ‘targeting HAP through multiple nursing care actions’ the participants suggested a variety of actions, that could be used as preventative measures for HAP, as part of nursing care. Participants acknowledged the importance of actions within several specific areas of nursing care: *respiration, mobility, oral health, and nutrition*. They also highlighted the importance of acting on these areas of care as a coordinated *interprofessional team*.

Participants stated that respiratory care targeting HAP often included breathing exercises. This was described as relying on multi-disciplinary teamwork, where members had different accountability. Some stated that it was common for both the RN and the physiotherapist (PT) to initiate and implement care actions such as these breathing exercises (Table 4, Q1). Others experienced the opposite, and described how the PT was mostly in charge of what should be done with the patient as part of respiratory care (Table 4, Q2). However, it was always the PT that assessed the degree of resistance, and determined what resistance equipment should be used for positive expiratory pressure (PEP) training, and follow-up procedures (Table 4, Q3). The RNs or the health care assistants were mainly involved in ensuring that the patients completed the exercises. The participants’ experiences of the usefulness of such PEP exercises in respiratory care targeting HAP varied. Some participants explained that the exercises were based on positive, proven experiences, while others were more sceptical and critical of the exercises, and shared experiences of implementation adherence failures when it came to supporting the patients during exercises (Table 4, Q3: Q4). The main reason given for failure was a lack of time, however participants also noted that the patients could not always perform the exercise, as they were sometimes complicated.

**Table 4:** The category, Targeting HAP through multiple nursing care actions

“… it is often the PT who prescribes. It is usually the PT who initiates it or the RN when he or she considers it as appropriate, but then it is us nurses, who will execute it.” (Q1, RN/B)	
“It is often them [The PT] approaching us, stating, ‘They should do the PEP training once an hour and they should be out of bed three times per 24 h for least 1 h.’ It is often they who prescribe what we should do with the patients.” (Q2, RN/D)	
“It is the PT who follows up on the PEP exercises, they are really good, and it works well, I think… I do it because I know by experience that it works, that it is good.” (Q3, RN/F)	
“Resistant breathing and stuff like that, it is a bit bad, I have to say, that it is not working optimally, but the PT might do it; otherwise, they have to have a schedule or demand that people should do it, but there is no time for that.” (Q4, RN/C)	
“To prevent pneumonia, first and foremost mobilisation is important so that the lungs are properly ventilated.” (Q5, RN/K)	
“I don’t maybe think that one has the focus exactly there at regular [that mobilisation of the bedridden patient] is about HAP; it is more pressure ulcers one think about.” (Q6, RN/F)	
“When the patients are reasonably stable, we try to position them at the bedside as early as possible, and we do have help from the PT and from the occupational therapist (OT). So, I think one is really good at trying to get them out of bed.” (Q7, RN/F)	
“I think that it is both the OT and the RN who see to it. The PT we have here has reasonably close communication, and I think that the OTs themselves come in and say that it is important that this patient gets out of bed.” (Q8, RN/D)	
“Over time the patient has developed HAP because one doesn’t have—I can’t say why—maybe lack of time, that one has not been able to mobilise the patient or because the patient is a ‘hoist’ patient with whom we deal at the end and then everything else happens during the day, so one forgets to take the patient out of bed.” (Q10, RN/G)	
“We put out the lists and tick off that oral care is done, maybe not as often as it should. Some hardly eat anything, and patients using a nasogastric tube should get oral care much more often. If a patient aspirate, there is a risk that the bacteria gets [down in the lungs]. So, it is really important with oral care for those patients.” (Q11, RN/L)	
“What I feel is that we miss a little; what we miss is actually oral care. There are bacteria in the oral cavity that are dangerous. One should be better at doing oral care before dysphagia assessment or when eating whatsoever.” (Q12, RN/A)	
“It is more about us doing oral care to avoid fungal infections, dryness, and rifts. I don’t believe that a lot of us address it. We have had lectures about what causes aspiration pneumonia, that it is bacteria in the oral cavity, but I do not know how many of us that consider that part.” (Q13, RN/B)	
“I make sure that the patients are sat up at mealtime, even if they are bedridden, so one must really follow up and almost position them in cardiac position so that they sit as good as possible.” (Q14, RN/G)	
“Sometimes, we do not have enough resources. Then, unfortunately, they are recumbent in bed and are getting fed there, and that is not the most optimal feeding situation one can experience, but we really try to raise them up as much as possible, we do.” (Q15, RN/L)	
“One learns a lot from each other, and it is facilitated by working evenings, weekends, and nights when [others on a multidisciplinary team] are not present but one has still learned a lot from them to build competence. Everyone has their part. I think it is really important, as then you get the best out of each of them.” (Q16, RN/A)	
“The speech therapists (ST) write their assessments so that we can read it, and they come back and [we] are given an oral report that recommends what the type of diet should be. So, we receive rather clear instructions. Then, it is always the medical doctor’s (MD) decision. We have a good relationship with the MDs, and we have a good team. So, if the RN says that this patient can’t swallow and the ST ordinates nil per mouth, then the patient gets an NGFT immediately.” (Q17, RN/E)	

Mobilisation was considered by some participants to be an important preventative measure in respiratory care. Other descriptions instead reflected the different levels of knowledge among the participants in regards to the preventative effects of mobilisation on HAP, or rather, their lack of knowledge of the relationship between a bedridden patient post stroke, and HAP risk (Table 4, Q5: Q6). The participants stated that a coordinated team was the cornerstone of respiratory care, and that the PT had a central role on the team when it concerned assessment and planning of a patient’s mobilisation. Participants also described how mobilisation was a routine part of nursing care at some stroke units (Table 4, Q7: Q8). Descriptions about less optimal mobility care were also present, with a lack of time again being cited as the chief reason for suboptimal care (Table 4, Q9).

In this category, oral health was described as another important part of respiratory care in the prevention HAP. Overall, the participants found that despite its importance, oral health care was not attended to as often as it should be, nor in an optimal way (Table 4, Q11: Q12). When oral health was part of routine care, it was described principally as being performed so that the patient could feel clean and comfortable. Thus, the relationship between oral health and HAP was not reflected in the participant’s understanding of their nursing practices (Table 4, Q13).

Nutrition was also highlighted as a vital part of respiratory care, particularly as many acute stroke patients suffer from dysphagia to varying degrees. Participants described some preventative measures around meals and mealtimes to reduce HAP risk (Table 4, Q14). Positioning and repositioning were described as a routine part of nursing care around mealtimes, regardless of whether the patients ate themselves, or using a NGFT. Lack of time and resources were stated to at times negatively impact respiratory nursing care, leading to bedridden patients not always being supported in an adequate feeding position at mealtimes (Table 4, Q15).

Lastly, the participant’s experiences highlighted the importance of multi-disciplinary teamwork. Teamwork was perceived as a key strength in care, particularly as working in multidisciplinary teams meant gaining more individual knowledge, in addition to support (Table 4, Q16). The multi-disciplinary team was described as a vital asset, particularly for patients with dysphagia, where collaboration was stated as being central to preventing the patient from swallowing incorrectly, which would increase the risk of HAP (Table 4, Q17).

### Challenges in providing respiratory care to patients in risk of HAP

In the category challenges in providing respiratory care to patients in risk of HAP, participants described some of the inherent trials faced in the identification and prevention of HAP. First, participants’ experiences reflected a lack of awareness regarding HAP. Additionally, care targeting HAP was *not a part of standard care or prioritised in nursing care* in the same way that assessing and evaluating routine health risk events were. Second, participants described a variety of *contextual barriers*, and at times, a general *lack of competence and knowledge* regarding the identification and prevention of HAP. On the one hand, general awareness of the risks of HAP was demonstrated by the participants, however, a lack of understanding relating to the risk factors leading to HAP was also found. Participants were able to describe the importance of identification and prevention of HAP, but this was not covered as a natural part of the RNs’ discussions about care (Table 5, Q1, Q2). The participants noted that while the identification and prevention of HAP was not a part of routine care (Table 5, Q3), their experiences were that some nursing interventions targeting HAP had been initiated. Notably however, participants also stated that such interventions were not a priority, and they believed that HAP could be prevented more effectively if relevant nursing care procedures were implemented (Table 5, Q4).

**Table 5:** The category, Challenges in providing respiratory care to patients in risk of HAP

“In general, around stroke, one talks about the risk of pneumonia, but I do not know to what degree we raise the issue or the question.” (Q1, RN/B)	
“Now, talking about [HAP], one realises that we are lacking. We do not have proper dialogues about [the risks], which is rather interesting. If one could discuss how to prevent it, it would have been a wake-up call for us.” (Q2, RN/A)	
“‘We do risk assessments on all patients for fall, nutrition, and pressure ulcers, one does care plans, and we have them on the board by the nursing station… but I have not yet seen one for pneumonia, and I think that would be good, I don’t know how, but [if we] had something for pneumonia, preventative like all the others, pressure ulcers, nutrition and falls.” (Q3, RN/K)	
“I think that most of us know in a way why we do these things, that it is important, but it is really easy to prioritise it down.” (Q4, RN/D)	
“I believe that if [we] could initiate all interventions and actions as we should, then we could prevent HAP. “I experience that we have too few staff to do it in a good way because there is a lot to be done when they arrive and sometimes many arrive on the same day. Some have had three red codes within half an hour with only two staff —one RN and one HCA — then it is really hard to find time for it all and do it well.” (Q5, RN/C)	
“The number of patients—instead of seven, one has 17—is far from optimal. And when one has more patients with multiple illnesses with more risk factors, there are also more problems, and unfortunately, the less control one has over keeping up. There were days when [one] did not get out.” (Q6, RN/H)	
“First of all, it is very medically oriented, if it is a bleed or to give thrombolytic treatment if it is an infarction. Then it might not be so much orientation towards nursing as it is about saving the brain.” (Q7, RN/B)	
“Particularly now when we have single rooms besides the observation room, it results in that one doesn’t see or interact with the patients in the same way as one does when patients are in a four- or two-bed room. If you went to one patient, you saw the others too.” (Q8, RN/A)	
“The speech therapist arrives and says, ‘He swallowed, so let’s follow this track now.’ However, it is seldom that the words preventing HAP is a part of it. But it feels like it is implicit and that everyone should understand that is why we do it. “If the ST says this is to prevent pneumonia, then [the RNs] maybe start to initiate other interventions, so we help each other. Instead of initiating one or two interventions, we can initiate three. We do initiate actions in the team, but we are not talking about why we are initiating them.” (Q9, RN/G)	
“I think that the challenge is staff turnover, the issue of competence, that you shift a lot, and [there] is a lot that one is expected to know.” (Q10, RN/B)	
“Yeah, maybe the one you work with doesn’t possess so much knowledge about how the patients can develop pneumonia, pressure ulcers, and all kind of complications. I think that this knowledge might be lacking among our staff.” (Q11, RN/K)	
“Alternatively, lack of competence, those with the [right] competence lacks time as they know everything that needs to be done. Those not really knowing might not understand this and don’t do as much. We who know what to do work ourselves to death.” (Q12, RN/C)	
“[They] can have everything, from a [slight] weakness in the hand to being completely paralysed, not being able to understand what I say or to make [themselves] understood. So, it is a lot one needs to think about. If one arrives as a new nurse to a patient with acute stroke, it is most certainly challenging.” (Q13, RN/F)	

Several contextual challenges were described as influencing the participants in the identification and prevention of HAP. The overall organisation of care at the different units i.e. staff numbers was one such challenge (Table 5, Q5). Another challenge for nursing care targeting HAP, was that participants experienced that they were put in charge of a larger number of complex patients than previously (Table 5, Q6), and that other medical activities and emergencies often took priority (Table 5, Q7). How the ward was configured was also stated to influence the number of direct nursing encounters that the patients received. This was described as negatively impacting the participants’ potential to assess and evaluate patients for HAP. Specifically, patients in single rooms were found by the participants to receive less attention than those patients who shared a room (Table 5, Q8).

Working in an multidisciplinary stroke team was also stated by the participants to be challenging at times. They described a somewhat ambiguous experience in relation to the coordinated teamwork regarding patients with dysphagia. This was because the focus often shifted primarily to single factors, for example, nutritional needs and swallowing exercises, without taking into considering the relationship between dysphagia and HAP (Table 5, Q9). In addition, participants also described how efficient and clear communication among the team could act as a ‘wake-up call’ for the RNs to initiate further actions targeting the prevention of HAP.

Participants also experienced additional challenges, including a general lack of competence and knowledge needed to identify and prevent HAP in their units. Staff turnover was one reason given for the difficulty in maintaining the relevant competence and knowledge base needed to care for stroke patients (Table 5, Q10: Q11). High turnover also meant that competence and knowledge was spread unevenly among the participants. Being a novice RN could mean that a participant more easily missed what was required to prevent HAP. This could in turn be experienced as putting additional strain on the more experienced RNs (Table 5, Q12). Participants also described how nursing care targeting HAP in this complex and high care-dependent group of patients, could be difficult for the less experienced RNs to undertake (Table 5, Q13).

## Discussion

The current study aimed to describe RNs’ experiences of providing respiratory care in relation to hospital acquired pneumonia (HAP) among patients with acute stroke being cared for at in-patient stroke units. Their experiences could be understood from the perspective of three overarching categories: (1) awareness of risk assessments and risk factors for HAP; (2) targeting HAP through multiple nursing care actions; and (3) challenges in providing respiratory care to patients in risk of HAP.

Identifying acute stroke patients at risk of HAP stood out as both complex and challenging. Being able to identify at-risk patients was considered an essential part of respiratory care, and demanded that the RNs were aware of a wide range of risk factors. Several specific risk factors were described, but dysphagia in particular was emphasised as being a key risk. The latter is in line with current evidence, suggesting that dysphagia constitutes one of the major risk factors for developing HAP [25–27]. The high awareness of this risk factor observed here, could be explained by that mandatory swallowing assessments included as a quality indicator in Riksstroke. Riksstroke is a tool aimed at continuous quality improvement in Swedish stroke care, as well as an instrument for following up the National Board of Health and Welfare’s guidelines for stroke care [28]. Although emphasised as a quality indicator, it was obvious that the adherence to standard practise, and the quality of swallow assessments performed, varied between stroke units. These findings imply that routine practises, and clearly defined standard quality indicators, can influence respiratory care. They also reflect individual differences among RNs, and within the multidisciplinary teams, which seem to affect adherence to standard practise doctrines, and their quality. It has previously been reported that when RNs have a positive attitude towards guidelines for the prevention of VAP, it influences their adherence to said guidelines [29]. This implies that interventions aimed at raising RN awareness of the importance of integrating evidence-based care routines are warranted. Further, raising awareness of the guidelines regarding swallowing assessments, to support the identification and prevention of HAP among patients with acute stroke, will be important.

One particularly noteworthy finding of this work, was that RNs did not associate the increased risk of developing HAP, and the recognised risk factor of poor oral health in relation to stroke. This is despite it being common knowledge that poor oral health is a risk factor in developing pneumonia [30, 31]. Instead, oral health was described as being part of routine care, but often-neglected. Interestingly, oral care was stated as primarily being undertaken for the patients’ general well-being; not for reducing the risk of developing HAP. This is corroborated by others, as through review, Ajwani and colleagues [32] found an international lack of oral health knowledge among RNs. They also highlighted infrequent assistance with stroke patient oral care. Overall, this stresses the need to incorporate oral hygiene and health care into the RNs’ responsibilities when delivering the fundamentals of care to acute stroke patients. This is especially vital in relation to respiratory care, as accumulated bacteria in the oral cavity due to neglected oral care, may increase the risk of HAP [33]. By utilising the dimension ‘integration of care needs’ in the fundamentals of care framework [11] as part of respiratory care, RNs may more easily understand the complex interactions between different risk factors for HAP.

At the four stroke units, that was part of this study, the identification of HAP was not described as part of standard care. Nevertheless, our findings described how the RNs conducted risk assessments and made care plans on a daily basis. These care plans mainly focused on the mandatory quality indicators outlined in Riksstroke, such as malnutrition, pressure ulcers, and falls. Focusing only on mandatory risk assessments does not constitute reasonable RN responsiveness to the needs of patients suffering from acute stroke. This may be the result from nursing care becoming more complex and technical, causing the fundamentals of nursing to be set aside [11, 34]. Such an explanation was partially corroborated by our findings, indicating that medically emergent activities, for example thrombolytic treatments, were often given higher priority than respiratory care per se.

Although preventative respiratory care to avoid HAP did not stand out as a standard clinical practise, our findings showed that when preventative nursing actions were conducted, it was not evident to the RNs that their actions could impact more than one of the known risk factors for HAP [27]. For example, the relationship between pneumonia and impaired mobility in relation to mobilisation and positioning [27]. It could be argued that RNs are expected to possess knowledge, competencies, and skills to plan preventative nursing care for HAP. In particular, this issue concerns RNs working in a stroke unit where HAP is not an unusual complication. Our findings indicate that further knowledge is needed among RNs about the development of safe and high-quality respiratory care for acute stroke and HAP. One way to achieve this, and to give respiratory care higher priority, may be to implement systematic and purposeful nursing rounds. Checking on patients at regular intervals can act as a primary procedure to address the patient’s essential personal needs [35, 36]. The fundamentals of care formulated by Kitson and colleagues [11] could be used to develop such a protocol, constituting the basis for observations during nursing rounds.

Earlier studies have shown that higher education levels, e.g., a Bachelor in nursing [37], and greater experience in working as an RN [17], are factors associated with a reduced incidence of complications such as pneumonia during hospitalisation. This is corroborated by our findings, in which RNs with a long work history in stroke care, and a higher educational level, appeared to better recognise and meet the complexities of the patients’ need for care. For less experienced RNs, it seemed like a challenge to assess, diagnose, intervene, and evaluate nursing care for patients with complex care needs. The respiratory care offered to the patients in relation to HAP seemed to be dependent on the individual RN’s competence, and therefore, was most likely inconsistent, and unequal. Consequently, higher priority should be given to respiratory care, as this will likely diminish the risk of HAP, resulting in faster rehabilitation trajectories, and shorter hospital stays for patients. One approach in the reduction of HAP incidence and improvement of respiratory care for patients with acute stroke, would be the implementation of evidence-based guidelines for the identification and prevention of HAP. Using such guidelines, routines, and care pathways can reduce the risk of HAP [38, 39]. Additionally, they increase the quality of nursing care, and address the diversity of care-related processes that are pivotal to diminishing risks [40], such as the possible impact of inconsistency in respiratory care.

Safe and high-quality care can only be achieved if the context in which RNs work is prepared to support nursing and nursing care. Our findings implied that the RNs paid less attention to direct patient care e.g., oral health, mobilisation and respiratory care. A possible explanation could be that they decided that such nursing care not was a priority. According to Maenhout and Vanhoucke [41], organisational structures and processes are known to affect the quality of care and the work environment of RNs. The inability to manage care effectively, threatens both the quality and safety of care [42]. Our findings also implied that a shortage of experienced nursing staff, high staff turnover, and patients’ more complex needs might explain the reality of a de-prioritisation of nursing care at some of these stroke units. The latter is corroborated by previous research [43], showing that similar barriers exist in preventing VAP. Indeed, nursing staff shortages have been described as an influential factor impeding the prevention of health-related complications such as pneumonia [38]. Additionally, to maintain a safe standard of care, it has been recommended that RNs are responsible for only two patients at a time during the acute phase of a stroke, and after that, only four patients [44]. In the current study, the RNs found that at times they did not actually have sufficient control over patient care to be able to identify HAP. For this reason, actual patient complexity and error margins to allow for complications and suboptimal timing must be considered when staffing is planned, rather than simply the number of patients that each RN is assigned responsibility for.

Modern stroke care is becoming increasingly centred around collaborative stroke units, this being a major factor in the improved number of stroke survivors [6]. The RNs in our study considered the multidisciplinary team a valuable resource in providing the respiratory care required to identify and prevent HAP. Nursing should be a part of multidisciplinary teams [14], but the RNs are ultimately responsible for the patients’ nursing care. Despite this, our findings implied that some of the responsibilities and initiatives for the patients’ care, such as mobilisation, positioning, and swallowing assessments, were left to the other members of the team. This result is corroborated by the findings of Clarke [16], in which RNs working in a rehabilitation stroke unit mainly focused on monitoring physical care, instead of integrating nursing with therapy work. Burton and colleagues [15] also found that no integration of therapist-initiated activities into fundamental aspects of nursing practice took place. They additionally noticed differences in therapeutic approaches, where RNs appeared safety driven, while the therapy staff stood out as much more comfortable with the notion of risk-taking [15]. To take the lead in care demands competent and knowledgeable RNs, however, at times our findings instead reflected a lack of knowledge and competency in the ability to identify and prevent HAP among acute stroke patients. The latter is especially concerning, considering the highly specialised environment that a stroke unit should reflect.

### Methodological considerations

Our qualitative descriptive study design meant that emphasis could be placed on the interpretation of participants’ experiences. This is particularly useful when the aim is to describe experiences unconditionally, or to find answers to the *what*, *how,* and *why* questions [18]. Using purposive sampling supported variation, as the participants worked at four stroke units, each representing different routines and organisations of care, which is likely to support the study’s generalisability [cf. 19]. Although the current study provides some valuable insight into RNs’ experiences of nursing care regarding post-stroke pneumonia, it is based on a small sample size (*n* = 11), which may have implications regarding its transferability. Nonetheless, securing large sample sizes is not a priority in qualitative study designs. Here, the focus lies instead on the included participants being able to provide different experiences of the phenomenon under study [45]. Guidance on the notion of response saturation was received after nine interviews, when no new information emerged from further interviews.

Using content analysis provided us with an opportunity to structure and present our findings according to three broad categories. However, there is always a risk of bias, i.e. subjectivity in data interpretation. It is therefore important to account for the researchers’ preconceptions, as these can influence both the questions posed during the interviews, and how the data are understood [21]. Two of the researchers (ME and MR) work within stroke care, while the other two (GB and MA) do not. Thus, to reduce the risk of subjectivity, and to improve the credibility of the current study, all authors took part in the development of the interview guide, as well as the analysis [21]. By describing the process of analysis and presenting quotations from the interviews (Table 3, 4 and 5), we aim to allow the reader to transparently judge our interpretations [19]. Qualitative studies such as this one are restricted in regard to transferability, as well as in applicability to other settings, and this needs to be considered when evaluating the findings.

## Conclusions and clinical implications

Respiratory care to prevent HAP among patients suffering from acute stroke is vital, however the findings from this study suggest that RNs may experience organisational challenges in providing such care. There is a need for increased knowledge relating to complex interactions in nursing, i.e., that some preventative nursing actions can influence more than one risk factor. For instance, poor oral health is associated with malnutrition and poor well-being, but also with an increased risk for HAP. Furthermore, procedures for identifying patients at risk of HAP need to be implemented, and RNs need better education in order to take ownership of respiratory nursing care within multidisciplinary teams. HAP is among the most common and serious respiratory complications experienced by patients with acute stroke. It causes distress, increased healthcare costs, morbidity, and even mortality. Respiratory care plays a vital role in the identification and prevention of HAP, but for it to be implemented successfully, RNs’ knowledge needs to be improved, the fundamentals of nursing care need to be prioritised, and evidence-based guidelines must be introduced. Moreover, RNs need to be offered further educational training, so that they can effectively lead nursing care in multidisciplinary stroke teams.

## Data Availability

The dataset from this study is available from the corresponding author upon reasonable request.

## Abbreviations

COREQ: Consolidated Criteria for Reporting Qualitative Studies
COPD: Chronic Obstructive Pulmonary Disease
ESO: European Stroke Organisation
HAP: Hospital Acquired Pneumonia
MD: Medical Doctor
NGFT: Naso Gastric Feeding Tube
OT: Occupational Therapist
PEP: Positive Expiratory Pressure
PT: Physio therapist
RN: Registered nurses
ST: Speech Therapists
VAP: Ventilator Associated Pneumonia

## References

[1] Hannawi Y, Hannawi B, Rao CPV, Suarez JI, Bershad EM (2013). Stroke-associated pneumonia: major advances and obstacles. Cerebrovasc Dis.

[2] Learoyd AE, Woodhouse L, Shaw L, Sprigg N, Bereczki D, Berge E, Caso V, Christensen H, Collins R, Czlonkowska A, El Etribi A, Farr TD, Gommans J, Laska AC, Ntaios G, Ozturk S, Pocock SJ, Prasad K, Wardlaw JM, Fone KC, Bath PM, Trueman RC, ENOS Trial investigators (2017). Infections up to 76 days after stroke increase disability and death. Transl Stroke Res.

[3] Finlayson O, Kapral M, Hall R, Asllani E, Selchen D, Saposnik G, Canadian Stroke Network; Stroke Outcome Research Canada (SORCan) Working Grou (2011). Risk factors, inpatient care, and outcomes of pneumonia after stroke. Neurology.

[4] Johnson W, Onuma O, Owalabi M, Sachdev S (2016). Stroke: a global response is needed. Bulletin of the World Health Organization.

[5] Langhorne, P., & Ramachandra, S. Organised inpatient (stroke unit) care for stroke: network meta-analysis. Cochrane Database of Systematic Reviews 2020, Issue 4. Art. No.:CD000197. DOI: 10.1002/14651858.CD000197.pub4. 10.1002/14651858.CD000197.pub4 PMC7197653 32324916

[6] Stroke Unit Trialists’ Collaboration. Organised inpatient (stroke unit) care for stroke. Cochrane Database of Systematic Reviews 2013, Issue 9. Art. No.: CD000197. DOI: 10.1002/14651858.CD000197.pub3. 10.1002/14651858.CD000197.pub3 PMC6474318 24026639

[7] Clarke DJ, Holt J (2015). Understanding nursing practice in stroke units: a Q-methodological study. Disabil Rehabil.

[8] de Jesus Oliveira I, Neves da Mota LA, Vaz Freitas S, Lopes Ferreira P (2019). Dysphagia screening tools for acute stroke patients available for nurses: A systematic review. Nursing Practice Today.

[9] Henderson V (1966). The nature of nursing; a definition and its implications for practice, research, and education.

[10] Richards DA, Borglin G (2019). ‘Shitty nursing’ - the new Normal? A Discussion Paper. Int J Nursing Studies.

[11] Kitson AL, Muntlin Athlin A, Conroy T (2014). Anything but basic: Nursing’s challenge in meeting patients’ fundamental care needs. J Nurs Scholarsh.

[12] Smith CJ, Kishore AK, Vail A, Chamorro A, Garau J, Hopkins SJ, Di Napoli M (2015). Diagnosis of stroke-associated pneumonia: recommendations from the pneumonia in stroke consensus group. Stroke..

[13] Reeves S, Pelone F, Harrison R, Goldman J, Zwarenstein M. Interprofessional collaboration to improve professional practice and healthcare outcomes. *Cochrane Database Systematic Reviews* PMID. 2017;28639262. 10.1002/14651858.CD000072.pub3 PMC6481564 28639262

[14] Cronenwett L, Sherwood G, Barnsteiner J, Disch J, Johnson J, Mitchell P, Sullivan DT, Warren J (2007). Quality and safety education for nurses. Nurses Outlook.

[15] Burton CR, Fisher A, Green TL (2009). The organisational context of nursing care in stroke units: a case study approach. Int J Nurs Stud.

[16] Clarke DJ (2014). Nursing practice in stroke rehabilitation: systematic review and meta-ethnography. J Clin Nurs.

[17] Jansson M, Ala-Kokko T, Ylipalosaari P, Syrjälä H, Kyngäs H (2013). Critical care nurses´ knowledge of, adherence to and barriers toward evidence-based guidelines for the prevention of ventilation-associated pneumonia - A survey study. Intensive Critical Care Nursing.

[18] Sandelowski M (2000). Whatever happened to qualitative description?. Res Nurs Health.

[19] Polit DF, Beck CT (2017). Nursing research. Generating and assessing evidence for nursing practice.

[20] Graneheim UH, Lundman B (2004). Qualitative content analysis in nursing research: concepts, procedures and measures to achieve trustworthiness. Nurse Educ Today.

[21] Graneheim UH, Lindgren B-M, Lundman U (2017). Methodological challenges in qualitative content analysis: a discussion paper. Nurse Educ Today.

[22] Tong A, Sainsbury P, Craig J (2017). Consolidated criteria for reporting qualitative research (COREQ): a 32-item checklist for interview and focus groups. Int J Qual Health Care.

[23] Krippendorff K (2013). Content analysis. An introduction to its methodology.

[24] Patton MQ (2002). Qualitative research and evaluation methods.

[25] Arnold, M., Liesirova, K., Broeg-Morvay, A., Meisterernst, J., Schlager, M., Mono, M.L., El-Koussy, M., Kägi, G., Jung S, & Sarikaya., H. (2016). Dysphagia in acute stroke: incidence, burden and impact on clinical outcome*.* PLoS One*,* 11(2), e0148424. doi: 10.1371/journal.pone.0148424. 10.1371/journal.pone.0148424 PMC4749248 26863627

[26] DiBardino D, Wunderink R (2015). Aspiration pneumonia: A review of modern trends. J Crit Care.

[27] Eltringham SA, Kilner K, Gee M, Sage K, Bray BD, Smith CJ, Pownall S. Factors associated with risk of stroke-associated pneumonia in patients with Dysphagia: A Systematic Review. Dysphagia. 2019. 10.1007/s00455-019-10061-6. 10.1007/s00455-019-10061-6 PMC7522065 31493069

[28] The National Board of Health and Welfare. (2020) National Guidelines for stroke care. [In Swedish]. Accessed 2020-09-21 at:https://www.socialstyrelsen.se/globalassets/sharepoint-dokument/artikelkatalog/nationella-riktlinjer/2020-1-6545.pdf.

[29] Kiyoshi-Teo H, Cabana MD, Froelicher ES, Blegen MA (2014). Adherence to institution-specific ventilator-associated pneumonia prevention guidelines. Am J Crit Care.

[30] Chick, A., & Wynne, A. (2020) Introducing an oral care assessment tool with advanced cleaning products into a high-risk clinical setting. *British Journal of Nursing*, 29(5):290-296. Doi:10.12968/bjon.2020.29.5.290. 10.12968/bjon.2020.29.5.290 32167815

[31] Pássaro L, Harbarth S, Landelle C (2016). Prevention of hospital- acquired pneumonia in non-ventilated adult patients: a narrative review. Antimicrob Resist Infect Control.

[32] Ajwani, S., Jayanti,S., Burkolter, N., Anderson, C., Bhole, S., Itaoui R., & George, A. (2017) Oral health care for stroke patients – a scoping review. J Clin Nurs, 26(7–8), 891–901. DOI: 10.1111/jocn.13520. 10.1111/jocn.13520 27538382

[33] Kwok C, McIntyre A, Janzen S, Mays R, Teasell R (2015). Oral care post stroke: a scoping review. J Oral Rehabil.

[34] Pipe TB, Connolly T, Spahr N, Lendzion N, Buchda V, Jury R, Cisar N (2012). Bringing Back the basics of nursing: defining patient care essentials. Nurs Adm Q.

[35] Mitchell MD, Lavenberg JG, Rebecca Trotta R, &. Umscheid, C.A. Hourly rounding to improve nursing responsiveness: A systematic review. J Nurs Adm. 2014;44(9):462–72. 10.1097/NNA.0000000000000101. 10.1097/NNA.0000000000000101 PMC4547690 25148400

[36] Shin N, Park J (2018). The effect of intentional nursing rounds based on the care model on Patients' perceived nursing quality and their satisfaction with nursing services. Asian Nursing Research.

[37] Aiken LH, Sloane DM, Bruyneel L, Van den Heede K, Griffiths P, Busse R, Diomidous M, Kinnunen J, Kózka M, Lesaffre E, McHugh MD, Moreno-Casbas MT, Rafferty AM, Schwendimann R, Scott PA, Tishelman C, van Achterberg T, Sermeus W, RN4CAST Consortium (2014). Nurse staffing and education and hospital mortality in nine European countries: a retrospective observational study. Lancet.

[38] Curtis LT (2008). Prevent of hospital-acquired infections: review of non- pharmacological interventions. J Hosp Infect.

[39] Meng K, Li Y, Li S, Zhao H, Chen L (2015). The survey on implementation of evidence- based nursing in preventing ventilator-associated pneumonia and the effect observation. Cell Biochem Biophys.

[40] Allen D (2019). Care trajectory management: A conceptual framework for formalizing emergent organisation in nursing practice. J Nurs Manag.

[41] Maenhout B, Vanhoucke M (2013). Analyzing the nursing organizational structure and process from a scheduling perspective. Health Care Management Science.

[42] Kobewka D, van Walraven C, Turnball J, Worthington J, Calder L, Forster A (2016). Quality gaps identified through mortality review. BMJ Quality & Safety.

[43] Atashi V, Yousefi H, Mahjobipoor H, Yazdannik A (2018). The barriers to the prevention of ventilator- associated pneumonia from the perspective of critical care nurses: A qualitative descriptive study. J Clin Nurs.

[44] Summers D, Leonard A, Wentworth D, Saver JL, Simpson J, Spilker JA, Hock N, Miller E, Mitchell PH, American Heart Association Council on Cardiovascular Nursing and the Stroke Council (2009). Comprehensive overview of nursing and interdisciplinary Care of the Acute Ischemic Stroke Patient. A scientific statement from the American Heart Association. Stroke.

[45] Marshall C, Rossman GB (2015). Designing qualitative research.

[46] World Medical Association (2013). World medical association declaration of Helsinki: ethical principles for medical research involving human subjects. J Am Med Assoc.

[47] Svensk Författningssamling, Lag (2004:406) om etikprövning av forskning som avser människor (In Swedish) Retrieved 2020-12-02 at https://www.riksdagen.se/sv/dokument-lagar/dokument/svensk-forfattningssamling/lag-2003460-om-etikprovning-av-forskning-som_sfs-2003-460.

[48] European Union. General Data Protection Regulation. The Regulation (EU) 2016/679.

